# 

**Published:** 1842-12

**Authors:** 


Volume III. DECEMBER, 1842. No. 2.

### THE AMERICAN JOURNAL AND LIBRARY OF Dental Science.

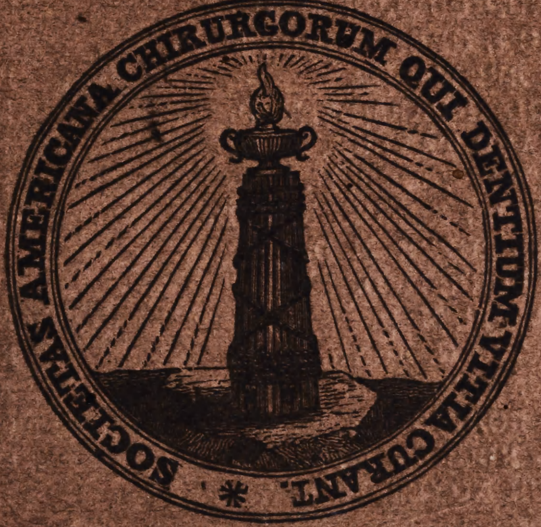

PUBLISHED UNDER THE AUSPICES OF THE

**American Society of Dental Surgeons.**

EDITORS:

CHAPIN A. HARRIS, M. D.

SOLYMAN BROWN, M.D.

LEONARD MACKALL, M. D.

NEW YORK:—GEORGE ADLARD,

BALTIMORE:—ARMSTRONG & BERRY,

LONDON:—SAMUEL HIGHLEY.

WOODS AND CRANE, PRS., BALT.

